# 977 Interdisciplinary Care Coordination to Maximize Rehabilitation Participation

**DOI:** 10.1093/jbcr/iraf019.508

**Published:** 2025-04-01

**Authors:** William Scott Dewey, Sarah Flores, Caroline Claassen, Adam Meyer, James Aden, Elisa Barboza, Barret Halgas, Leopoldo Cancio

**Affiliations:** United States Army Institute of Surgical Research Burn Center; United States Army Institute of Surgical Research Burn Center; United States Army Institute of Surgical Research Burn Center; United States Army Institute of Surgical Research Burn Center; United States Army Institute of Surgical Research Burn Center; United States Army Institute of Surgical Research Burn Center; United States Army Institute of Surgical Research Burn Center; United States Army Institute of Surgical Research Burn Center

## Abstract

**Introduction:**

Rehabilitation in the acute care setting can have a significant impact on functional outcomes and is recognized as part of the Intensive Care Unit liberation bundle. A previous performance improvement project in 2018 found that wound care and wound assessments were the most common barriers to rehabilitative care in our burn center. An interdisciplinary approach to care coordination was needed to optimize rehabilitation participation without compromising wound care and assessments.

**Methods:**

Several interdisciplinary meetings were held to identify process issues and ascertain solutions to optimize rehabilitation care delivery. Changes that were discussed included the following:

1. Limiting the number of patients down for wound assessment early in the morning

2. Assigning scheduled rehabilitation sessions early in the morning in lieu of wound assessments

3. Medical staff performing wound evaluations later in the day

4. Identifying patient care sequencing for the following day during multidisciplinary rounds

5. Posting visual aids to depict the planned patient care sequences for the following day

Staff members were educated on the above changes before initiation. A tracking system was used to record the number of missed treatment sessions monthly. The May 2024 baseline dataset of missed treatments due to wound care or assessments was compared to the post-intervention months of June-August. Data were analyzed by chi square tests.

**Results:**

Missed treatments as a percentage of those planned were as follows:

• May: 28/596 (4.7%)

• June: 8/715 (1.1%)

• July: 10/625 (1.6%)

• August: 3/554 (0.5%)

(Pre vs. post intervention, p < 0.001)

Monthly comparison of baseline vs. post-implementation data showed a reduction in missed treatments each month:

• May vs. June: 71% (p < 0.001)

• May vs. July: 64% (p < 0.001)

• May vs. August: 89% (p = 0.002)

**Conclusions:**

We achieved a substantial reduction in monthly missed treatments for 3 consecutive months through improved coordination. These findings suggest interdisciplinary collaboration can be effective in reducing barriers to increase patients’ rehabilitation participation.

**Applicability of Research to Practice:**

Conflicting care priorities among disciplines is a common problem in burn centers. This effort demonstrates collaboration can be an effective means to address these competing interests.

**Funding for the Study:**

N/A

